# Supplementary material for: Quantification of FRET-induced angular displacement by monitoring sensitized acceptor anisotropy using a dim fluorescent donor
Source: Nat Commun. 2021 May 5;12:2541. doi: 10.1038/s41467-021-22816-7 (PMC8099864; doi:10.1038/s41467-021-22816-7)
Supplement: Supplementary file 2 — Supplementary Information [file 41467_2021_22816_MOESM2_ESM.pdf]

Supplementary Information

Quantification of FRET-induced angular displacement by monitoring sensitized acceptor anisotropy using a dim fluorescent donor

Danai Laskaratou[1], Guillermo Solís Fernández[2], Quinten Coucke[2], Eduard Fron[2,3], Susana Rocha[2], Johan Hofkens[2], Jelle Hendrix[2,4], Hideaki Mizuno[1*]

[1] Laboratory for Biomolecular Network Dynamics, Biochemistry, Molecular and Structural Biology Section, Department of Chemistry, KU Leuven, 3001 Heverlee, Belgium
[2] Chem&Tech-Molecular Imaging and Photonics, Department of Chemistry, KU Leuven, 3001 Heverlee, Belgium
[3] KU Leuven Core Facility for Advanced Spectroscopy, KU Leuven, 3001 Heverlee, Belgium
[4] Dynamic Bioimaging Lab, Advanced Optical Microscopy Centre and Biomedical Research Institute, Hasselt University, Agoralaan C (BIOMED), B-3590 Diepenbeek, Belgium
* e-mail address: hideaki.mizuno@kuleuven.be

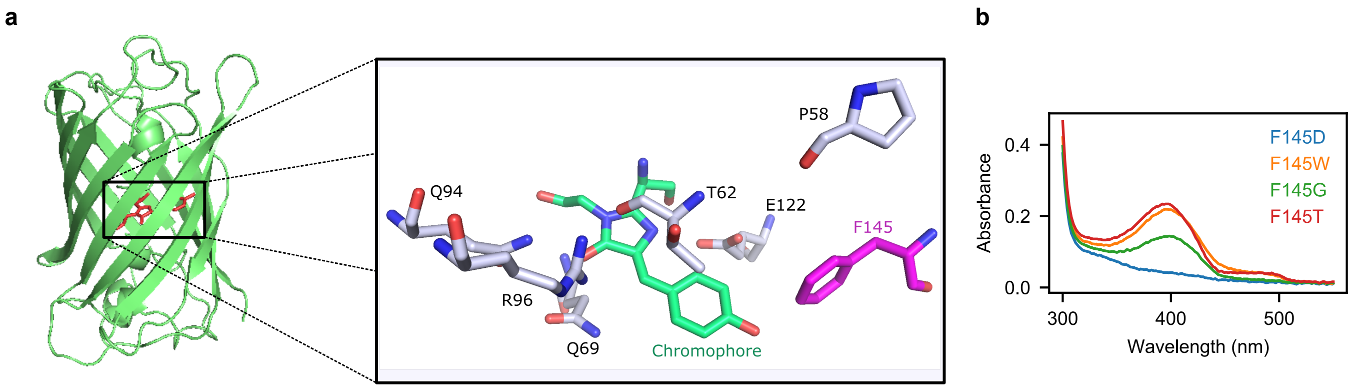

Supplementary Fig. 1|Sapphire structure, chromophore, and mutations at position 145 a. Model structure based on crystal structure of wtGFP (pdb id: 1GFL). All H9-40 (Sapphire) mutations (T203I, S72A, Y145F) were manually introduced to the wtGFP structure. The chromophore and its surrounding environment are shown in the zoomed-in black box. The chromophore is highlighted in green and the strategic residue F145 in magenta. **b.** Absorption spectra from crude extract of Sapphire-F145D, -F145W, -F145G, and -F145T. Source data are provided as a Source Data file.

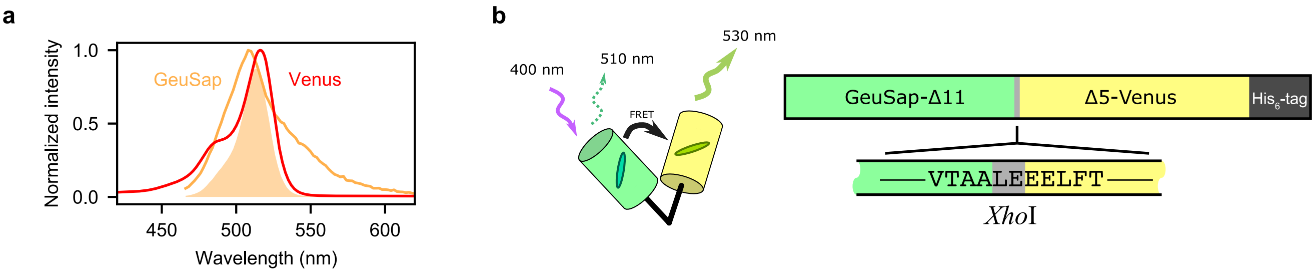

Supplementary Fig. 2|Tandem construct GeuSV11.5. a. Normalized emission of GeuSap and absorption of Venus. The shaded part represents the overlap area. **b.** Cartoon representation (left) and protein structure (right) of the tandem construct GeuSV11.5. Source data are provided as a Source Data file.

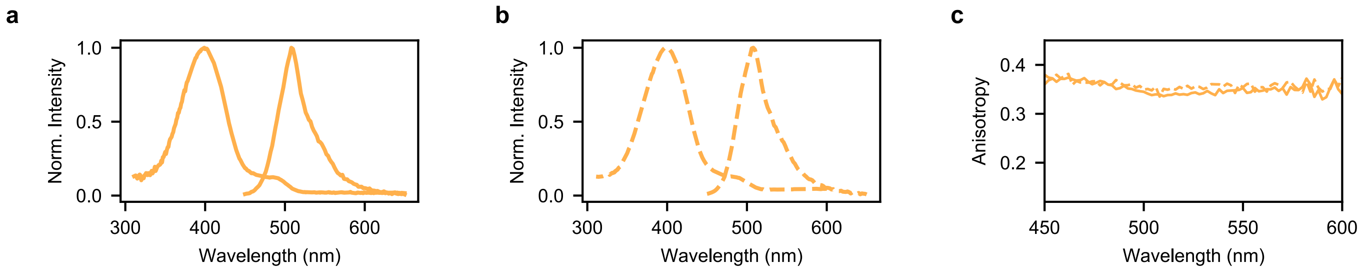

Supplementary Fig. 3|Spectral comparison between GeuSap and GeuSapA206K. a. Normalized absorption and emission spectra of GeuSap. **b.** Normalized absorption and emission spectra of GeuSapA206K. **c.** Steady-state anisotropy vs emission of GeuSap (solid line) and GeuSapA206K (dashed line). Excitation was at 400 nm. Source data are provided as a Source Data file.

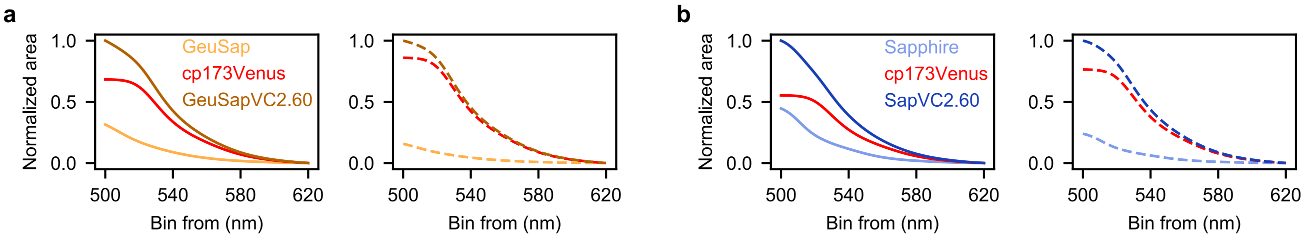

Supplementary Fig. 4|Cumulative distribution analysis of GeuSapVC2.60 and SapVC2.60 per detection range. a. Complementary cumulative distribution analysis of GeuSapVC2.60 in Ca^{2+}-free (left panel) and Ca^{2+}-saturated conditions (right panel). The analysis was performed as follows: after linear unmixing, the resolved signals were cumulatively integrated using the composite trapezoidal rule (by going gradually from 620 nm to 500 nm). Then, the GeuSapVC2.60 signal was normalized to 1 at the maximum value corresponding to range of 620-500 nm), and resolved signals of donor and acceptor were multiplied by the same normalization factor. **b.** Same analysis performed for SapVC2.60 in Ca^{2+}-free (left panel) and Ca^{2+}-saturated conditions (right panel). Source data are provided as a Source Data file.

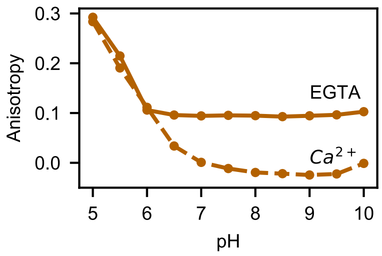

Supplementary Fig. 5|pH titration of GeuSapVC2.60 in solution. Titration curves obtained in the presence of EGTA (100 μM) and Ca^{2+} (1 mM). Excitation was at 405 nm and detection at 530-560 nm. From the collected images, anisotropy was calculated as described in the Materials and Methods. Source data are provided as a Source Data file.

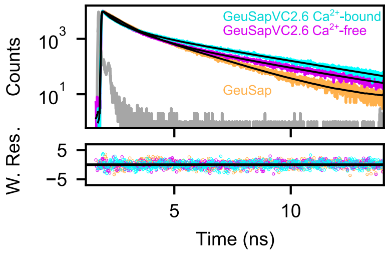

Supplementary Fig. 6|Fluorescence decays of GeuSap, GeuSapVC2.60 Ca^{2+}-free and -bound forms in solution. All decays were obtained by 400 nm excitation and 510 nm detection by TCSPC. For GeuSapVC2.60, measurements were performed in the presence of EGTA (100 μM) or Ca^{2+} (1 mM). The decays were fitted to multi-exponential models with reconvolution (for fitting parameters refer to Supplementary Table 4). W. Res.: weighted residuals. Source data are provided as a Source Data file.

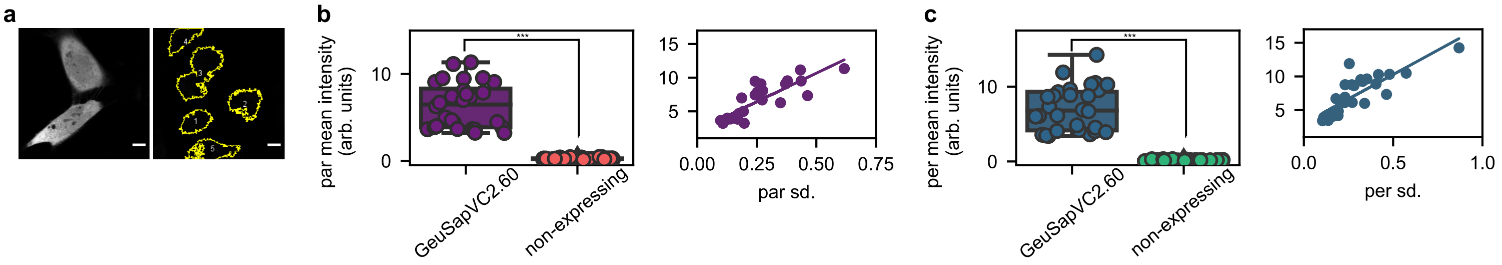

Supplementary Fig. 7|Analyses of HeLa cells transfected with GeuSapVC2.60 or non-transfected. a. Typical fluorescnce image of HeLa cells transfected with GeuSapVC2.60 (left) and blank (non-transfected) (right). Data were acquired under the same settings and each image was produced by summing 40 frames (100 s) from parallel channel. Then the same lookup table was used for representation. The yellow traces in the right image indicate the shape of cells outlined. Imaging of transfected cells was repeated at least 10 times, yielding basically the same results. For non-transfected cells we only used one dish, where we imaged 6 different fields of view with at least 7 cells per field, yielding basically the same results. Scale bar is 10 μm. **b.** Box plots of parallel channel mean intensity (40 frames, 100 s) for transfected (N=28) and non-transfected (N=41) HeLa cells (left). SIgnificance was tested with t-test (two-sided, no equal population variance assumed), and stars represent $p=1x10^{-12}$. Linear regression of parallel channel mean intensity (signal) versus standard deviation (noise) (right). The slope corresponding to signal-to-noise ratio was 17±2. **c.** Same type of analysis for perpendicular channel. $p=1.1x10^{-12}$. Signal-to-noise ratio was 14±2. All data in this figure were collected before histamine treatment. For boxplots in panels b, c: center line, median; box limits, upper and lower quartiles; whiskers, minimum and maximum. Source data are provided as a Source Data file.

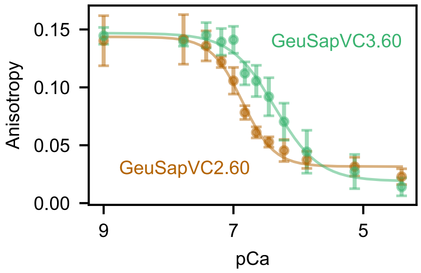

Supplementary Fig. 8| *In situ* Ca^{2+} titration of Geuda Cameleons. HeLa cells were transfected with GeuSapVC2.60 or GeuSapVC3.60 and incubated in buffers with varying Ca^{2+} concentrations in the presence of 4-bromo-A23187, an ionophore which allows the equilibration of Ca^{2+} levels across the cell membrane. Cells were imaged after each buffer addition. Fitting with Hill function yielded n=1.8±0.2 and K'_d=(138±11) nM for GeuSapVC2.60, and n=1.3±0.2 and K'_d=(436±55) nM for GeuSapVC3.60. These values are essentially the same as the ones obtained *in vitro*, with the exception of GeuSapVC2.60, which is 1.9 times higher. This is known in the literature, where sensors expressed in cells tend to show slightly higher K'_d values compared to *in vitro*[1]. The obtained fitted values of minimum and maximum anisotropy were r_{max}=0.14±0.00 and r_{min}=0.03±0.00 for GeuSapVC2.60, and r_{max}=0.15±0.00 and r_{min}=0.02±0.01 for GeuSapVC3.60. In both cases these values are shifted up compared to the *in vitro* experiment, which is consistent with a higher viscosity environment in the cell. More than 3 cells were imaged for each point. Data points and error bars represent mean values ± SD. Source data are provided as a Source Data file.

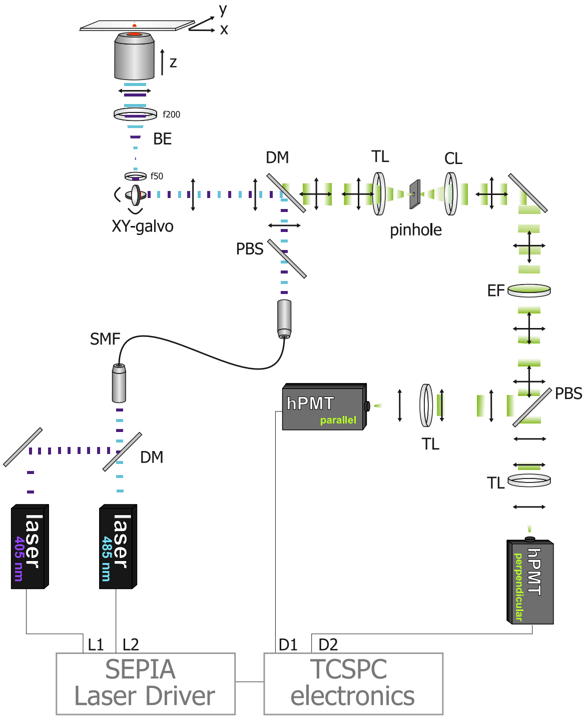

Supplementary Fig. 9|Home-built laser-scanning confocal microscope. SMF: single-mode optical fiber, PBS: polarizing beam splitter, DM: dichroic mirror, BE: beam expander, TL: tube lens, CL: collimating lens, EF: emission filter, hPMT: hybrid photomultiplier tube.

Supplementary Table 1|Photophysical properties of Sapphire-F145 mutants.

Sample	ε_{400} ($M^{-1}cm^{-1}$)	Lifetimes (ns)		QY
		$\tau_1(a_1)$	$\tau_2(a_2)$	
WT	$3.5x10^4$	3.3 (1)	-	0.64[2]
F145S	$2.9x10^4$	2.9 (1)	-	0.41
F145T	$3.3x10^4$	2.8 (1)	-	0.50
F145G	$3.4x10^4$	2.7 (1)	-	0.47
F145W	$3.4x10^4$	0.58 (0.7)	1.7 (0.3)	0.12

Supplementary Table 2|Excited-state kinetics of GeuSap.

Sample	$k_r(s^{-1})$	$k_{nr}(s^{-1})$	Proper folding ratio (%)
Sapphire	$1.9x10^8$	$1.1x10^8$	57
GeuSap	$1.3x10^8$	$9.7x10^8$	54

Supplementary Table 3|Fluorescence decay fitting parameters of GeuSap and GeuSV11.5 at 400 nm excitation.

Sample (detection wavelength)	Lifetimes (ns) $\tau_1(a_1)$	$\tau_2(a_2)$	$\tau_3(a_3)$	τ_d (ns)[*]
GeuSap (510)	0.58 (0.7)	1.7 (0.3)	-	0.92
GeuSV11.5 (510)	0.06 (0.55)	0.66 (0.2)	3.0 (0.25)	0.22[**]
GeuSV11.5 (530)	0.15 (0.23)	2.8 (0.37)	3.4 (0.4)	-

[*]amplitude-weighted average donor lifetime

[**]τ_3 was eliminated from the calculation, assuming it is an acceptor component

Supplementary Table 4|Fluorescence decay fitting parameters of GeuSap, GeuSapVC2.60 Ca^{2+}-free and Ca^{2+}-bound at 400 nm excitation and 510 nm detection.

Sample	Lifetimes (ns)			τ_d (ns)[*]
	$\tau_1(a_1)$	$\tau_2(a_2)$	$\tau_3(a_3)$	
GeuSap	0.58 (0.7)	1.7 (0.3)	-	0.92
GeuSapVC2.60 Ca^{2+}-free	0.27 (0.46)	0.87 (0.42)	2.8 (0.12)	0.55[**]
GeuSapVC2.60 Ca^{2+}-bound	0.21 (0.5)	0.8 (0.33)	3.1 (0.17)	0.44[**]

[*]amplitude-weighted average donor lifetime

[**]τ_3 was eliminated from the calculation, assuming it is an acceptor component

Supplementary Table 5|Binding affinities of GeuSapVC Ca^{2+} sensors.

Sample	K_d (nM)	Hill coefficient	Anisotropy	
			r_{min}	r_{max}
GeuSapVC2.6	71±3	1.9±0.2	-0.03	0.10
GeuSapVC3.6	(5.0±0.1)x10^2	1.6±0.1	-0.02	0.10

Supplementary Table 6|Fluorescence properties of A206K mutants.

Sample	ε_{400} (M^{-1} cm^{-1})	QY
Sapphire	$3.5x10^4$	0.64
$Sapphire_{A206K}$	$3.4x10^4$	0.65
GeuSap	$3.4x10^4$	0.12
$GeuSap_{A206K}$	$3.2x10^4$	0.14

Supplementary Table 7|List of primers used in this study.

Name	Sequence
QC_aqGFPF145W_fw	GGAGTACAACTGGAACAGCCACAAC
QC_aqGFPA206K_fw	GAGCTACCAGTCCAAACTGAGCAAAGACC
QC_aqGFPF145S_fw	GCTGGAGTACAACTCCAACAGCCACAA
QC_aqGFPF145D_fw	GCTGGAGTACAACGACAACAGCCACAACG
QC_aqGFPF145G_fw	GCTGGAGTACAACGGCAACAGCCACAACG
QC_aqGFPF145T_fw	GCTGGAGTACAACACCAACAGCCACAACG
aqGFP_BamHIfrGAT_fw	GAAGAAGGATCCGATGGTGAGCAAGGGCGAG
aqGFPd11_XhoI_rev	GGCCGGCTCGAGGGCGGCGGTCACGAACTC
aqGFPd5_XhoI_fw	GGCCGGCTCGAGGAGGAGCTGTTCACCGGG
aqGFP*_HindIII_rev	GAAGAAAAGCTTCGAATTACTTGTACAGCTCGTCCATGCC
aqGFP_NcoI_frATG_fw	GAAGATCCATGGTGAGCAAGGGCG
aqGFPd11_SphI_rev	GAAGAAGCATGCAGGCGGCGGTCACG
cp173V_XhoI_rev	GAAGAACTCGAGCCCCTCGATGTTGTGGCGGATC
CaM_SphI_fw	GAAGAAGCATGCATGACCAACTGAC
cp173V*_EcoRI_rev	GAAGAAGAATTCTTACTCGATGTTGTGGCGGATC
aqGFP_HindIII_fw	GAAGAAAAGCTTATGGTGAGCAAGGGCG

Supplementary Information References

1. Thomas, D., Tovey, S. C., Collins, T. J., Bootman, M. D., Berridge, M. J., & Lipp, P. (2000). A comparison of fluorescent Ca^{2+} indicator properties and their use in measuring elementary and global Ca^{2+} signals. Cell calcium, 28(4), 213-223.

2. Tsien, R. Y. (1998). The green fluorescent protein. Annual review of biochemistry, 67(1), 509-544.
