# Supplementary material for: Quantification of FRET-induced angular displacement by monitoring sensitized acceptor anisotropy using a dim fluorescent donor
Source: Nat Commun. 2021 May 5;12:2541. doi: 10.1038/s41467-021-22816-7 (PMC8099864; doi:10.1038/s41467-021-22816-7)
Supplement: Supplementary file 5 — Reporting Summary [file 41467_2021_22816_MOESM5_ESM.pdf]

nature research

Corresponding author(s): Prof. Hideaki Mizuno

Last updated by author(s): Mar 19, 2021

Reporting Summary

Nature Research wishes to improve the reproducibility of the work that we publish. This form provides structure for consistency and transparency in reporting. For further information on Nature Research policies, see our Editorial Policies and the Editorial Policy Checklist.

Statistics

For all statistical analyses, confirm that the following items are present in the figure legend, table legend, main text, or Methods section.

n/a	Confirmed	
☐	☒	The exact sample size (n) for each experimental group/condition, given as a discrete number and unit of measurement
☒	☐	A statement on whether measurements were taken from distinct samples or whether the same sample was measured repeatedly
☐	☒	The statistical test(s) used AND whether they are one- or two-sided<br>*Only common tests should be described solely by name; describe more complex techniques in the Methods section.*
☒	☐	A description of all covariates tested
☒	☐	A description of any assumptions or corrections, such as tests of normality and adjustment for multiple comparisons
☐	☒	A full description of the statistical parameters including central tendency (e.g. means) or other basic estimates (e.g. regression coefficient) AND variation (e.g. standard deviation) or associated estimates of uncertainty (e.g. confidence intervals)
☐	☒	For null hypothesis testing, the test statistic (e.g. F, t, r) with confidence intervals, effect sizes, degrees of freedom and P value noted<br>*Give P values as exact values whenever suitable.*
☒	☐	For Bayesian analysis, information on the choice of priors and Markov chain Monte Carlo settings
☒	☐	For hierarchical and complex designs, identification of the appropriate level for tests and full reporting of outcomes
☒	☐	Estimates of effect sizes (e.g. Cohen's d, Pearson's r), indicating how they were calculated

Our web collection on statistics for biologists contains articles on many of the points above.

Software and code

Policy information about availability of computer code

Data collection	Data was collected with the following software: FluorEssence v 3.5 (Horiba Fluorolog 3), SPCM v 9.77 (Becker & Hickl), Olympus FluoView FV1000 software v 4.2c, SymPhoTime 64 (PicoQuant).
Data analysis	Initial image processing and analysis were performed using ImageJ (v 1.52p), Matlab (r2018b), and PAM (v 1.2) (http://www.gitlab.com/PAM-PIE/PAM). Fitting of fluorescence decays was performed with TRFA software (v 1.4). Further analysis was performed with home-written Python scripts. The scripts are available upon request.

For manuscripts utilizing custom algorithms or software that are central to the research but not yet described in published literature, software must be made available to editors and reviewers. We strongly encourage code deposition in a community repository (e.g. GitHub). See the Nature Research guidelines for submitting code & software for further information.

Data

Policy information about availability of data

All manuscripts must include a data availability statement. This statement should provide the following information, where applicable:

- Accession codes, unique identifiers, or web links for publicly available datasets
- A list of figures that have associated raw data
- A description of any restrictions on data availability

Raw data for all experiments can be found at https://doi.org/10.6084/m9.figshare.14248487.v2

Field-specific reporting

Please select the one below that is the best fit for your research. If you are not sure, read the appropriate sections before making your selection.

[x] Life sciences   [ ] Behavioural & social sciences   [ ] Ecological, evolutionary & environmental sciences

For a reference copy of the document with all sections, see nature.com/documents/nr-reporting-summary-flat.pdf

Life sciences study design

All studies must disclose on these points even when the disclosure is negative.

Sample size	More than 20 cells were analyzed for each sensor for calcium imaging.
Data exclusions	No data was excluded from analyses.
Replication	Imaging experiments in living cells were repeated at least 10 times and produced essentially same results. Representative results are shown in the manuscript. For in situ titration we performed the experiment once and analyzed between 3-6 cells for every experimental point, and the result was consistent with in vitro titration.
Randomization	For calcium imaging we only used two sensors and did imaging in random order spanning several weeks. For in situ titration there is no way to randomize, since different buffers are added sequentially to the same sample.
Blinding	This study includes no experiments that require blinding tests to avoid any biases. Measurements were performed exactly the same way, data acquired under exactly the same settings, and plotted using the same scripts.

Reporting for specific materials, systems and methods

We require information from authors about some types of materials, experimental systems and methods used in many studies. Here, indicate whether each material, system or method listed is relevant to your study. If you are not sure if a list item applies to your research, read the appropriate section before selecting a response.

Materials & experimental systems

n/a	Involved in the study	
[x]	[ ]	Antibodies
[ ]	[x]	Eukaryotic cell lines
[x]	[ ]	Palaeontology and archaeology
[x]	[ ]	Animals and other organisms
[x]	[ ]	Human research participants
[x]	[ ]	Clinical data
[x]	[ ]	Dual use research of concern

Methods

n/a	Involved in the study	
[x]	[ ]	ChIP-seq
[x]	[ ]	Flow cytometry
[x]	[ ]	MRI-based neuroimaging

Eukaryotic cell lines

Policy information about cell lines

Cell line source(s)	HeLa cells were from ATCC (CCL-2).
Authentication	Cell morphology and growth were checked regularly by visual inspection under the microscope.
Mycoplasma contamination	The cell lines were not tested for mycoplasma contamination.
Commonly misidentified lines (See ICLAC register)	No commonly misidentified cell lines were used.
