# Supplementary material for: Quantification of FRET-induced angular displacement by monitoring sensitized acceptor anisotropy using a dim fluorescent donor
Source: Nat Commun. 2021 May 5;12:2541. doi: 10.1038/s41467-021-22816-7 (PMC8099864; doi:10.1038/s41467-021-22816-7)
Supplement: Supplementary file 6 — Description of Additional Supplementary Files [file 41467_2021_22816_MOESM6_ESM.pdf]

Title: Supplementary Movie 1:
Description: Ca^{2+} imaging in HeLa cells with GeuSapVC2.60. The movie is in IMD mode. Color hue indicates anisotropy. Scale bar is 10 µm.

Title: Supplementary Movie 2:
Description:Ca^{2+} imaging in HeLa cells with GeuSapVC3.60. The movie is in IMD mode. Color hue indicates anisotropy Scale bar is 10 µm.
